# Mulberry flavonoids modulate rumen bacteria to alter fermentation kinetics in water buffalo

**DOI:** 10.7717/peerj.14309

**Published:** 2022-12-14

**Authors:** Mengwei Li, Faizul Hassan, Lijuan Peng, Huade Xie, Xin Liang, Jiaxiang Huang, Feng Huang, Yanxia Guo, Chengjian Yang

**Affiliations:** 1 Key Laboratory of Buffalo Genetics, Breeding and Reproduction Technology, Guangxi Buffalo Research Institute, Chinese Academy of Agricultural Sciences, Nanning, Guangxi, China; 2 Institute of Animal and Dairy Sciences, University of Agriculture, Faisalabad, Faisalabad, Punjab, Pakistan

**Keywords:** Mulberry flavonoids, Rumen bacteria, Predicted metagenome, Functional profile, Rumen fermentation, Water buffalo

## Abstract

Mulberry flavonoids can modulate the composition of rumen microbiota in ruminants to improve nutrient digestibility, owing to their strong biological activities. This study aimed to explore the effect of mulberry leaf flavonoids (MLF) on rumen bacteria, fermentation kinetics, and metagenomic functional profile in water buffalo. Forty buffaloes (4 ± 1 lactations) with almost same body weight (av. 600 ± 50 Kg) and days in milk (90 ± 20 d) were randomly allocated to four treatments having different levels of MLF: 0 g/d (control), 15 g/d (MLF15), 30 g/d (MLF30), and 45 g/d (MLF45) supplemented in a basal diet. After 35 days of supplementation, rumen contents were collected to determine rumen fermentation parameters. The 16S rRNA gene sequencing was performed to elucidate rumen bacteria composition. The obtained taxonomic data were analyzed to explore the rumen bacteriome and predict the associated gene functions and metabolic pathways. Results demonstrated a linear increase (*p* < 0.01) in rumen acetate, propionate, and total VFAs in the MLF45 group as compared to control. No effect of treatment was observed on rumen pH and butyrate contents. Acetate to propionate ratio in the MLF45 group linearly and quadratically decreased (*p* = 0.001) as compared to MLF15 and control groups. Similarly, MLF45 linearly increased (*p* < 0.05) the microbial protein (MCP) and NH_3_-N as compared to other treatments. Treatment adversely affected (*p* < 0.01) almost all alpha diversity parameters of rumen bacteria except Simpson index. MLF promoted the abundance of Proteobacteria while reducing the relative abundances of Actinobacteria, Acidobacteria, Chloroflexi, and Patescibacteria. The MLF supplementation tended to substantially reduce (0.05 < *p* < 0.1) the abundance of Actinobacteria, and Patescibacteria while completely eliminating Acidobacteria (*p* = 0.029*)*, Chloroflexi *(p* = 0.059*)*, and Gemmatimonadetes (*p* = 0.03) indicating the negative effect of flavonoids on the growth of these bacteria. However, MLF45 tended to substantially increase (*p* = 0.07) the abundance (~21.5%) of *Acetobacter*. The MLF treatment exhibited negative effect on five genera by significantly reducing (Sphingomonas) or eliminating (Arthobactor, unclassified_c__Actinobacteria, norank_c__Subgroup_6, norank_o__Saccharimonadales, and Nocardioides) them from the rumen microbiota. Pearson correlation analysis revealed 3, 5 and 23 positive correlations of rumen bacteria with milk yield, rumen fermentation and serum antioxidant parameters, respectively. A positive correlation of MCP was observed with three bacterial genera (Acetobacter, Enterobacter, and Klebsiella). The relative abundance of Pseudobutyrivibrio and Empedobacter also showed a positive correlation with the ruminal acetate and propionate. The present study indicated 45 g/d as an appropriate dose of MLF which modulated rumen bacteria and its functional profile in water buffalo.

## Introduction

The quest for natural feeds, fodders and phytochemical compounds is increasing tremendously to replace antibiotic growth promoters in livestock feeding (El-Zaiat & Abdalla, 2019; Sallam et al., 2018). In this regard, fodders and shrubs with higher polyphenols particularly flavonoid contents are increasingly important as they not only improve nutrient digestibility but also alleviate oxidative stress and reduce methane emission (CH_4_) from ruminants (El-Zaiat et al., 2020; Morsy et al., 2021). Plant flavonoids are a diverse group of plant secondary compounds ubiquitously found in different plant species throughout the plant kingdom. Flavonoids are known as benzol-pyrone with potent anti-inflammatory, antioxidant, and antimicrobial properties (Balcells et al., 2012; Polumackanycz et al., 2019). Flavonoids possess significant antimicrobial activities as they can inhibit the activity of various gram-negative and gram-positive bacteria as well as protozoa through disruption of cell integrity (Xie et al., 2015). Besides, a large number of studies have demonstrated antibacterial and antiprotozoal effects of propolis flavonoids (de Aguiar Sílvia et al., 2013). Among plant secondary metabolites, flavonoids from mulberry leaf possess significant antioxidant potential. Owing to their excellent antioxidant activities, mulberry leaf flavonoids (MLF) are of great importance from the biological and pharmacological point of view. Numerous studies have confirmed the antioxidant capacity of mulberry leaves or their extract in rats, cattle, and sheep (Peng et al., 2018; Cirne et al., 2019).

Rumen microbiome is responsible for the major physiological activities related to nutrient digestion and utilization. Owing to diverse antimicrobial activity, flavonoids possess promising potential to alter the population dynamics of rumen microbes. Therefore, flavonoids can potentially modulate the rumen microbiota leading to subsequent changes in nutrient digestion and metabolism. Alfalfa flavonoids have shown to promote the population of cellulolytic bacteria (*Butyrivibrio fibrisolvens*) in dairy cows (Zhan et al., 2017). Interestingly, flavonoids also possess the ability to alter rumen fermentation dynamics but also favor beneficial microbes like *M. elsdenii* (lactate-utilizing bacteria), which can subsequently impart desirable effects on animal performance (Seradj et al., 2014). In addition, flavonoids have also shown direct toxic effects on methanogens and protozoa to reduce methanogenesis in the rumen (Patra & Saxena, 2010). Like other plant flavonoids, MLF have also shown excellent antioxidant and anti-inflammatory activities (Chon et al., 2009) coupled with their effective ability to improve nutrient digestibility and reduce methane emission in ruminants (Chen et al., 2016). In addition to their antioxidant and other biological activities, MLF have also shown to exert desirable effects on rumen function to sustain animal health and performance (Besle et al., 2010; Stoldt et al., 2016). Despite the potent abilities of MLF, no previous study has reported their potential effects on the abundance and diversity of rumen bacteria and rumen fermentation parameters in buffalo.

Our companion study evaluated the effect of dietary supplementation of MLF on the milk yield and composition of buffaloes during the summer season (Li et al., 2020). The findings revealed that MLF supplementation effectively alleviated the heat stress while increasing daily milk yield, fat corrected milk, and milk protein content. Moreover, MLF treatment also substantially promoted the levels of serum metabolic hormones, including growth hormone, prolactin, and estradiol in lactating buffaloes. Based on these findings, it was hypothesized that increasing concentrations of MLF can directly influence rumen microbiome composition and fermentation parameters which would result in better digestibility and nutrient absorption leading to superior performance in terms of increased milk yield and milk protein content in buffaloes. Therefore, effect of MLF supplementation on the diversity of rumen bacteria, rumen fermentation parameters and functional profile was further explored in the present study. For this experiment, we used the same buffaloes of our previous study by collecting rumen fluid at the end of the experiment for the determination of rumen fermentation and bacterial diversity parameters (Li et al., 2020).

## Materials and Methods

### Mulberry leaf flavonoids

Mulberry leaf extract was purchased from a commercial company (Xi’an Feida Biotechnology Co. Ltd., Xi’an, China) having 5% flavonoid content that mainly constituted 65% flavones, 20% flavonols, and 15% other polyphenols.

### Experimental design and animal management

Ethics committee of the Chinese Academy of Agriculture Sciences, Guangxi Buffalo Research Institute, China approved all experimental procedure of the present study (Approval No. BRI-2019010). This study was conducted from June to July 2019 at the Guangxi Buffalo Research Institute, located in Nanning, South China (N 22° 53′ 22.59″N, E 108° 21′ 51.19′′E). Murrah buffaloes belonging to the Guangxi Buffalo Research Institute, Nanning were enrolled for this study. Guangxi Buffalo Research Institute maintains a largest buffalo herd in China having different breeds of buffaloes including Nili-Ravi, Murrah, Mediterranean, and Swamp buffalo breeds. Forty lactating Murrah buffaloes were randomly selected for this study keeping in view of similar parity (av. 4 ± 1), stage of lactation (90 ± 20 d) and average body weight (600 ± 50 Kg). Randomization in allotment of treatments were done through drawing the numbered cards. Details of meteorological data, physiological and milk performance recording of buffaloes have been described in our companion study (Li et al., 2020). Buffaloes were fed with total mix ration (TMR) consisting of grass (*Pennisetum purpureum schum*), brewer’s grain, and corn concentrate mixture for 5 weeks. According to different doses of MLF supplementation, four treatment groups (10 buffaloes per group) were designed: MLF15 (15 g/d/head), MLF30 (30 g/d/head), MLF45 (40 g/d/head) and control group (0 g/d/head). Proximate analysis of TMR was performed to determine dry matter (DM), crude protein (CP), and ash content using standard procedures (Association of Official Analytical Chemists (AOAC), 1999). Neutral detergent fiber (NDF) and acid detergent fiber (ADF) were determined using an ANKOM2000 Fiber Analyzer Unit (ANKOM Technology Corp., Macedon, NY, USA) using alpha-amylase and sodium sulfite, respectively (Association of Official Analytical Chemists (AOAC), 1999; Soest, Robertson & Lewis, 1991). The chemical composition of basal diet (TMR) is presented in Table S1.

### Collection of rumen fluid and determination of rumen fermentation parameters

After 5 weeks of supplementation, rumen contents were collected before morning feeding from experimental buffaloes using an oral stomach tube (OST). The OST used in this study was designed and manufactured by Anscitech Co. Ltd. (Wuhan, China). Compared to other methods of rumen sampling, the OST is considered as a simple, quick and less invasive way of rumen sampling (Duffield et al., 2004) and has been used extensively for collection of ruminal fluid (Lodge-Ivey, Browne-Silva & Horvath, 2009). About 500 mL of rumen contents were collected in sterilized plastic bottles and put on ice, then immediately transported to the lab for further analysis. Rumen contents were strained using cheesecloth to remove big feed particles and collect particle-associated rumen liquor according to the previous report (Zebeli et al., 2008). After the collection of particle-associated rumen liquor, pH was immediately measured using a pH meter.

An aliquot of rumen liquor was acidified with an equivalent volume (4 mL) of 0.2 M HCl and stored at −20 °C for the determination of ammonia-N (NH_3_-N) by indophenols method (Weatherburn, 1967). Briefly, 5 mL filtrate was centrifuged (1,000 rpm) for 5 min at 4 °C and supernatant was collected. After that collected supernatant (1.5 mL) was centrifuged (12,000 rpm at 4 °C) for 15 min to pellet the microbial cells. Afterward, 0.5 mL of (0.25N) NaOH was added to the pelleted microbial cells followed by thorough mixing. Then it was incubated at 100 °C in a water bath for 20 min. After that it was again centrifuged for 30 min (12,000 rpm at 4 °C) to collect supernatant for analysis of microbial CP using the colorimetric method by observing absorbance at 595 nm through spectrophotometer (721 spectrophotometer colorimeter) using bovine serum albumin solution (1 mg/mL) as a standard equivalent (Makkar et al., 1982). Samples of VFA fractions (C2, C3, C4, C5, iC4, and iC5) were measured using the GC system, as described previously (Qin, 1982).

### DNA extraction and 16S rRNA gene sequencing

DNA from rumen contents was extracted using the CTAB bead beating method as reported previously (Yu & Morrison, 2004). The quality of DNA was checked on the NanoDrop spectrophotometer (NanoDrop2000; Thermo Scientific, Waltham, MA, USA).

Illumina MiSeq sequencing was carried out after library preparation from purified DNA using barcoded primers for the V3-V4 region of the 16S rRNA gene (Klindworth et al., 2013). DNA libraries were sequenced using a 2 × 300 paired-end sequencing module (Illumina, San Diego, CA, USA). Optimization and quality control of sequence reads was performed using FLASH and Trimmomatic software. After performing quality control, taxonomic assignment was performed to identify Operational Taxonomic Units (OTUs) by sequence alignment against the SILVA (Release128) and RDP Classifier databases, as reported previously (Quast et al., 2012). After identification of OTUs, an abundance of OTU was used to generate an OTU table followed by a grouping of OTUs at each phylogenetic level. The Qiime software was used to conduct all the above-mentioned steps regarding taxonomic assignment of rumen bacteria as described previously (Caporaso et al., 2010).

Both alpha and beta diversity parameters were analyzed using complete OTU table to explore differences in bacterial diversity in different treatment groups. Bacterial richness and evenness in each sample were analyzed by measuring Chao, ACE (Abundance based coverage estimator) while Shannon and Simpson indices were measured to estimate alpha diversity (Lee, 1992; Shannon, 1948). Meanwhile, microbial evenness within each sample was assessed by Simpson and Shannon’s evenness (Pielou’s J) indices (Smith & Wilson, 1996). The differences caused by treatment in bacterial diversity were measured by beta diversity by determining Bray-Curtis dissimilarities across different treatment groups using PERMANOVA (999 permutations) as described previously (Bray & Curtis, 1957; Anderson, 2001).

### Metagenome prediction and functional profiling

Functional annotations of prokaryotic genomes were predicted by analysis of 16S rRNA gene sequences using the Tax4FunR package coupled with SILVA database (Aßhauer & Meinicke, 2013). This predicted metagenome was used to obtain gene ontologies and metabolic pathways based on the KEGG database, as previously reported (Biscarini et al., 2018; Kanehisa et al., 2016).

### Statistical analysis

The effect of MLF on rumen fermentation and bacterial alpha diversity was analyzed using the PROC GLM procedure of SAS (SAS Institute Inc., Cary, NC, USA) having treatment as a fixed effect and animal as a random effect. Significant means were compared using Duncan’s multiple range test. Relative abundance of rumen bacteria at phyla and genera level was compared using the Kruskal-Wallis H test with a false discovery rate (FDR) correction and Tukey-Kramer as a *post-hoc* test to elucidate differences across treatment groups. Results were considered significant at *p* < 0.05 while tendency was considered at 0.05 < *p* < 0.1. Pearson correlation coefficients (r) were measured with the vegan package of R software (R 3.2) to determine the potential relationship rumen bacteria with different rumen fermentation parameters. Moreover, correlation of rumen bacteria with milk yield/composition and serum antioxidant parameters including malondialdehyde (MDA), total antioxidant capacity (T-AOC), superoxide dismutase (SOD), catalase (CAT), and glutathione peroxidase (GSH-Px) recorded in our previous study was also investigated (Li et al., 2019). The correlation matrix was visualized using the pheatmap package of R software by displaying a two-dimensional heat map. The defined color and its strength show the type of correlation (nature and strength, respectively). Asterisk sign was used when the r value was greater than 0.1 and the *p* values were less than 0.05 (*0.01 < *p* ≤ 0.05, **0.001 < *p* ≤ 0.01, ***p ≤ 0.001).

## Results

### Rumen fermentation parameters

MLF supplementation had significant effect on almost all rumen fermentation parameters except pH and concentration of butyrate which showed no change as compared to the control (Table 1). MLF45 treatment linearly increased (*p* < 0.01) the concentration of rumen acetate and propionate as compared to control. However, there was no significant difference in acetate and propionate concentration among the other three groups. MLF45 linearly and quadratically decreased the acetate to propionate ratio (*p* = 0.001) as compared to MLF15 and control, while showed no significant difference when compared with MLF30. Linear increase (*p* = 0.004) in total VFAs was observed in the MLF45 group as compared to other groups. Likewise, MLF45 linearly increased (*p* < 0.05) the concentrations of ruminal MCP and NH_3_-N as compared to other groups.

**Table 1:** Effects of Mulberry leaf flavonoids on different rumen fermentation parameters in buffalo.

Parameter	Control	MLF15	MLF30	MLF45	SEM	*p* Value		
						Treat	Linear	Quadratic
Acetate (mmol/L)	13.47^b^	15.27^ab^	14.98^ab^	17.38^a^	0.79	0.047	0.012	0.714
Propionate (mmol/L)	9.47^b^	8.98^b^	11.46^b^	14.83^a^	0.85	0.005	0.001	0.053
Acetate/Propionate	1.42^b^	1.70^a^	1.30^bc^	1.17^c^	0.04	0.001	1.000	0.001
Butyrate (mmol/L)	4.44	5.45	5.14	5.24	0.54	0.595	0.406	0.425
Total VFA (mmol/L)	27.39^b^	29.70^b^	31.59^ab^	37.46^a^	1.82	0.023	0.004	0.357
pH	6.59	6.73	6.59	6.46	0.15	0.683	0.471	0.394
NH3-N (mg/dL)	9.27^b^	8.82^b^	10.76^ab^	11.28^a^	0.58	0.049	0.015	0.426
MCP (mg/dL)	14.08^b^	14.83^b^	16.19^ab^	17.12^a^	0.64	0.040	0.006	0.894

**Note:**

Values with different superscripts (a,b,c) in the same row indicate significant differences between treatments (*p* < 0.05)

### Rumen bacterial diversity

#### Taxonomic statistics

**Figure 1:**
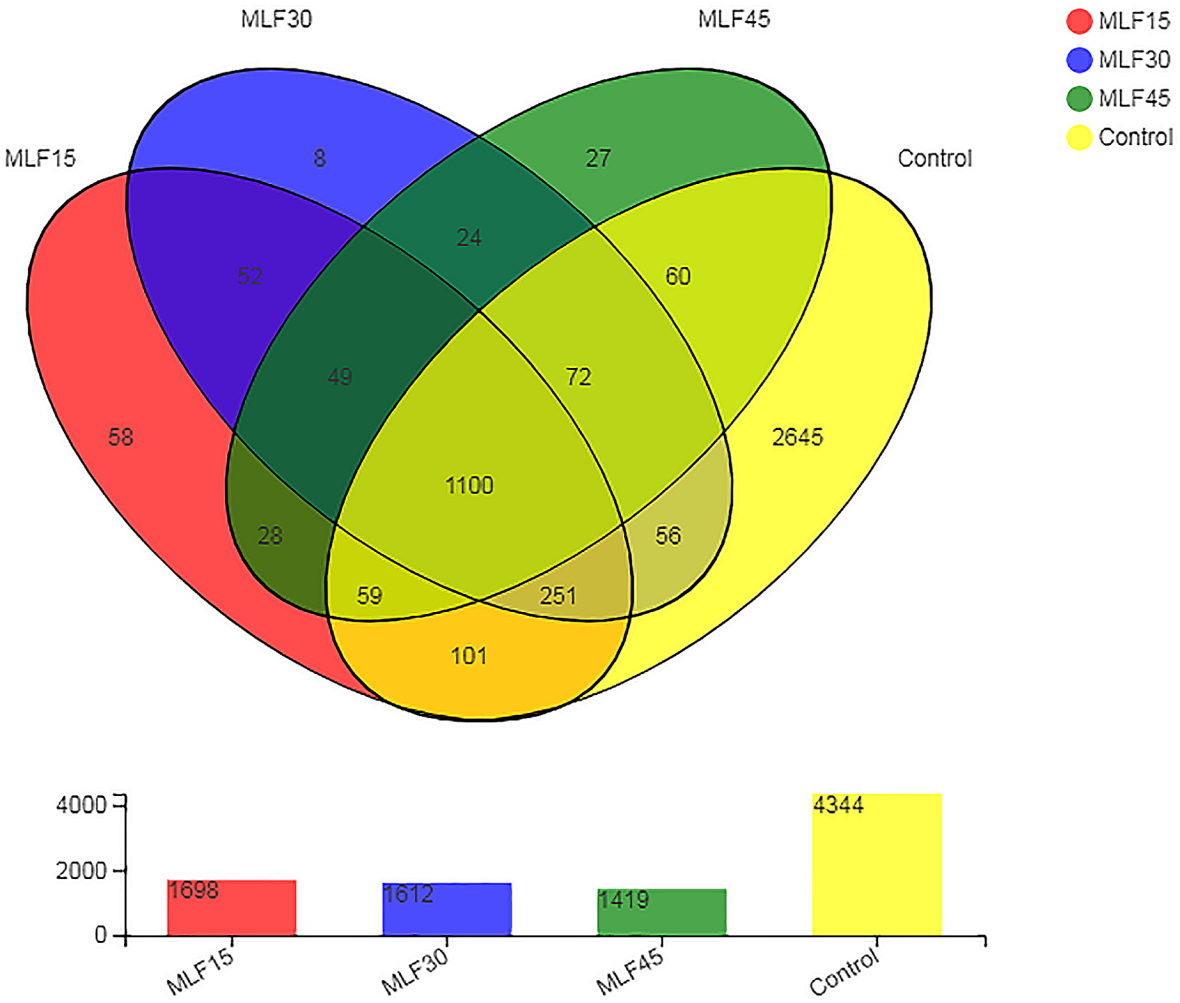
Distribution of unique and shared OTUs across different treatment groups.

Analysis of the 16S rRNA gene sequence data revealed a total of 4,590 OTUs in all rumen fluid samples. After quality control, these OTUs were classified into 38 phyla, 99 classes, 255 orders, 435 families, 885 genera, and 1722 species of rumen bacteria. The distribution of shared and unique OTUs among different treatment groups is shown in Fig. 1. The greatest numbers of OTUs (4,344) were observed in control as MLF supplementation linearly decreased the number of OTUs. The lowest OTU number was observed in MLF45 (1,419) followed by MLF 30 (1,612) and MLF15 (1,698). The 1100 OTUs were similar in all groups, while the rest of the OTUs (2,738) were unique with respect to different groups. The highest number of unique OTUs was observed in the control (2,645) followed by MLF15 (58), MLF45 (27), and MLF30 (eight) groups.

### Alpha diversity parameters

Treatment significant affected all alpha diversity parameters except the Simpson index (Table 2). A linear and quadratic (*p* = 0.001) decrease was observed in number of observed species (sobs), ace, and Chao indices of alpha diversity. Likewise, MLF linearly (*p* < 0.01) decreased the Shannon index and evenness while quadratically (*p* = 0.004) decreased the Simpson evenness.

**Table 2:** Effect of MLF supplementation on Alpha diversity parameters of rumen bacteria.

Parameter	Control	MLF15	MLF30	MLF45	SEM	*p* Value		
						Treat	Linear	Quadratic
sobs	3024^a^	1293^b^	1179^b^	1027^b^	58.481	0.001	0.001	0.001
shannon	6.11^a^	5.23^b^	5.18^b^	4.45^c^	0.137	0.001	0.001	0.586
simpson	0.0086	0.0184	0.0138	0.0709	0.019	0.14	0.059	0.238
ace	3278.49^a^	1533.49^b^	1427.09^bc^	1227.06^c^	72.07	0.001	0.001	0.001
chao	3261.45^a^	1546.85^b^	1450.52^bc^	1244.24^c^	70.355	0.001	0.001	0.001
shannoneven	0.763^a^	0.730^a^	0.734^a^	0.643^b^	0.019	0.01	0.003	0.16
simpsoneven	0.0393^bc^	0.0448^ab^	0.0619^a^	0.0215^c^	0.006	0.007	0.189	0.004

**Note:**

Values with different superscripts (a,b,c) in the same row indicate significant differences between treatments (*p* < 0.05).

### Relative abundance of bacterial phyla and genera

**Figure 2:**
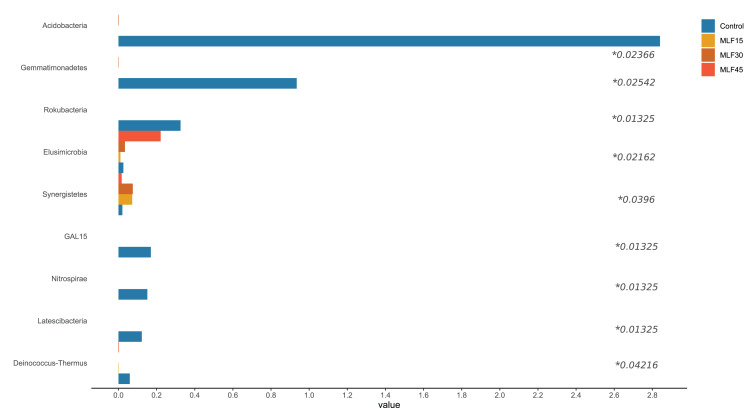
Relative abundance of bacterial phyla across different treatment groups. An asterisk (*) indicates the significant difference (*p* < 0.05) in the relative abundance of respective bacterial taxa.

There were significant shifts in rumen bacteria in response to MLF supplementation (Fig. 2, Table S2). Three major bacterial phyla, including *Bacteroidetes, Firmicutes*, and *Proteobacteria*, were observed in buffalo rumen. The relative abundance of *Proteobacteria* was almost two-fold higher (*p* = 0.07) in the MLF45 group than that in control. However, the other two levels of MLF supplementation favored (*p* > 0.05) Bacteroidetes and Firmicutes as compared to the control group. Interestingly, the abundance of Actinobacteria, and Patescibacteria was substantially reduced (0.05 < *p* < 0.1) with MLF supplementation while completely eliminating Acidobacteria (*p* = 0.029*)*, Chloroflexi *(p* = 0.059*)*, and Gemmatimonadetes (*p* = 0.03) indicating the negative effect of flavonoids on the growth of these bacteria (Fig. 2). In addition, MLF treatment also exhibited negative effect on Rokubacteria, GAL15, Nitrospirae, Latescibacteria, and Deinococcus-Thermus by eliminating (*p* < 0.05) them from rumen microbiota (Fig. 2), however, relative abundance of these phyla was <1% of the total sequences. Similarly, two minor phyla Elusimicrobia and Synergistetes (<1% abundance) were positively affected by MLF treatment (*p* < 0.05).

In fact, five phyla, including Bacteroidetes, Firmicutes, Proteobacteria, Acidobacteria, and Actinobacteria, accounted for 90% of total rumen bacteria detected in the present study.

**Figure 3:**
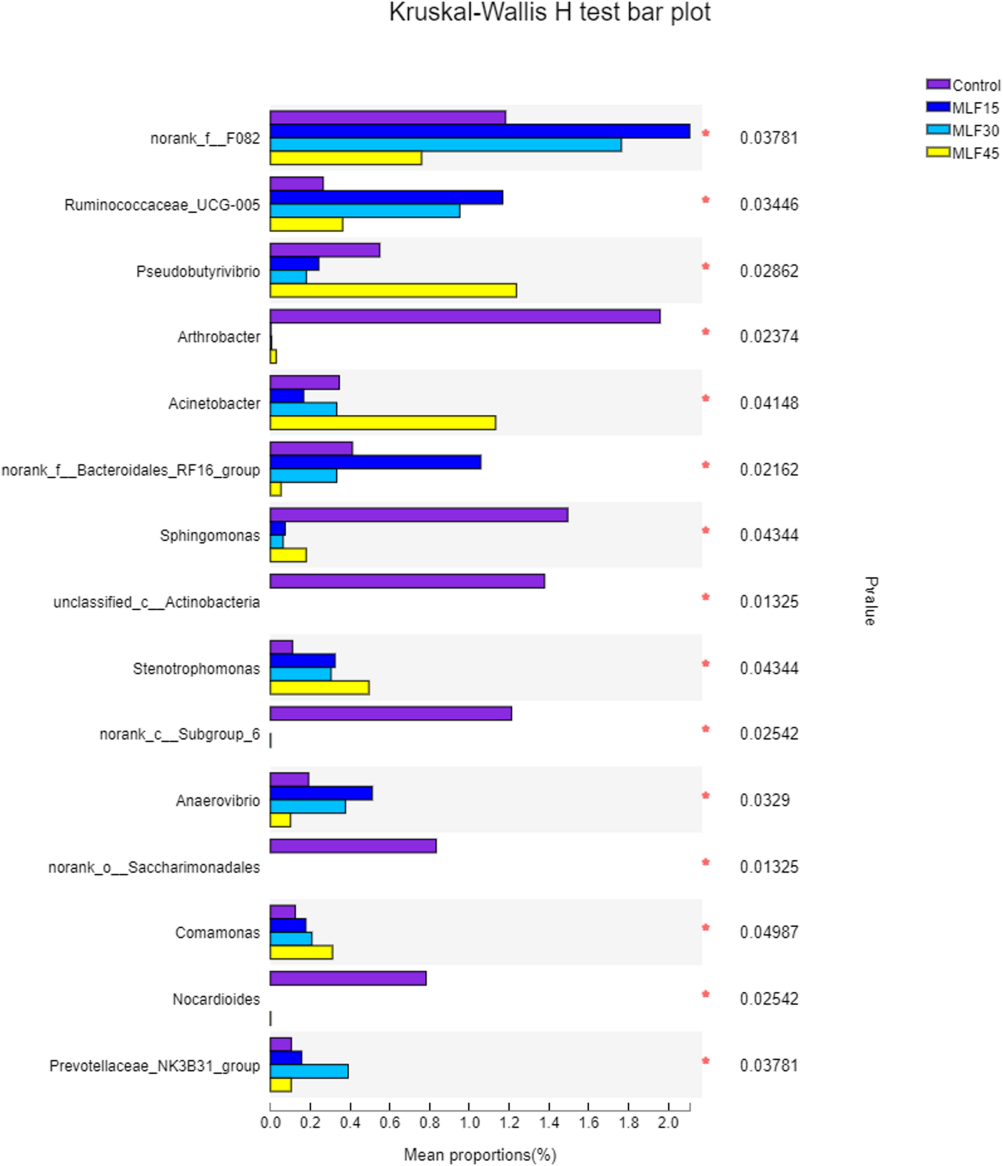
Relative abundance of bacterial genera across different treatment groups. An asterisk (*) indicates the significant difference (*p* < 0.05) in the relative abundance of respective bacterial taxa.

The relative abundance of *Prevotella* increased (*p* = 0.16) both in MLF15 and MLF30 while decreased in the MLF45 as compared to the control group (Table S3). However, the *Acetobacter* increased both in MLF15 and MLF45 groups, with a marked tendency for increase (5.5 fold) in the latter group (*p* = 0.07). The MLF15 group showed a higher abundance (*p* < 0.05) of four bacterial genera, including norank_f__F082, Ruminococcaceae_UCG-005, norank_f__Bacteroidales_RF16_group and Anaerovibrio than the other groups. Interestingly, MLF45 led to a higher abundance of *Pseudobutyrivibrio* (*p* = 0.03) and Acinetobactor (*p* = 0.04) than groups, revealing the favorable effect of higher dose of flavonoids on these bacterial genera. The MLF treatment exhibited negative effect on five genera by significantly reducing (Sphingomonas) or eliminating (Arthobactor, unclassified_c__Actinobacteria, norank_c__Subgroup_6, norank_o__Saccharimonadales, and Nocardioides) them from the rumen microbiota (Fig. 3).

### Association of rumen bacteria with rumen fermentation parameters

**Figure 4:**
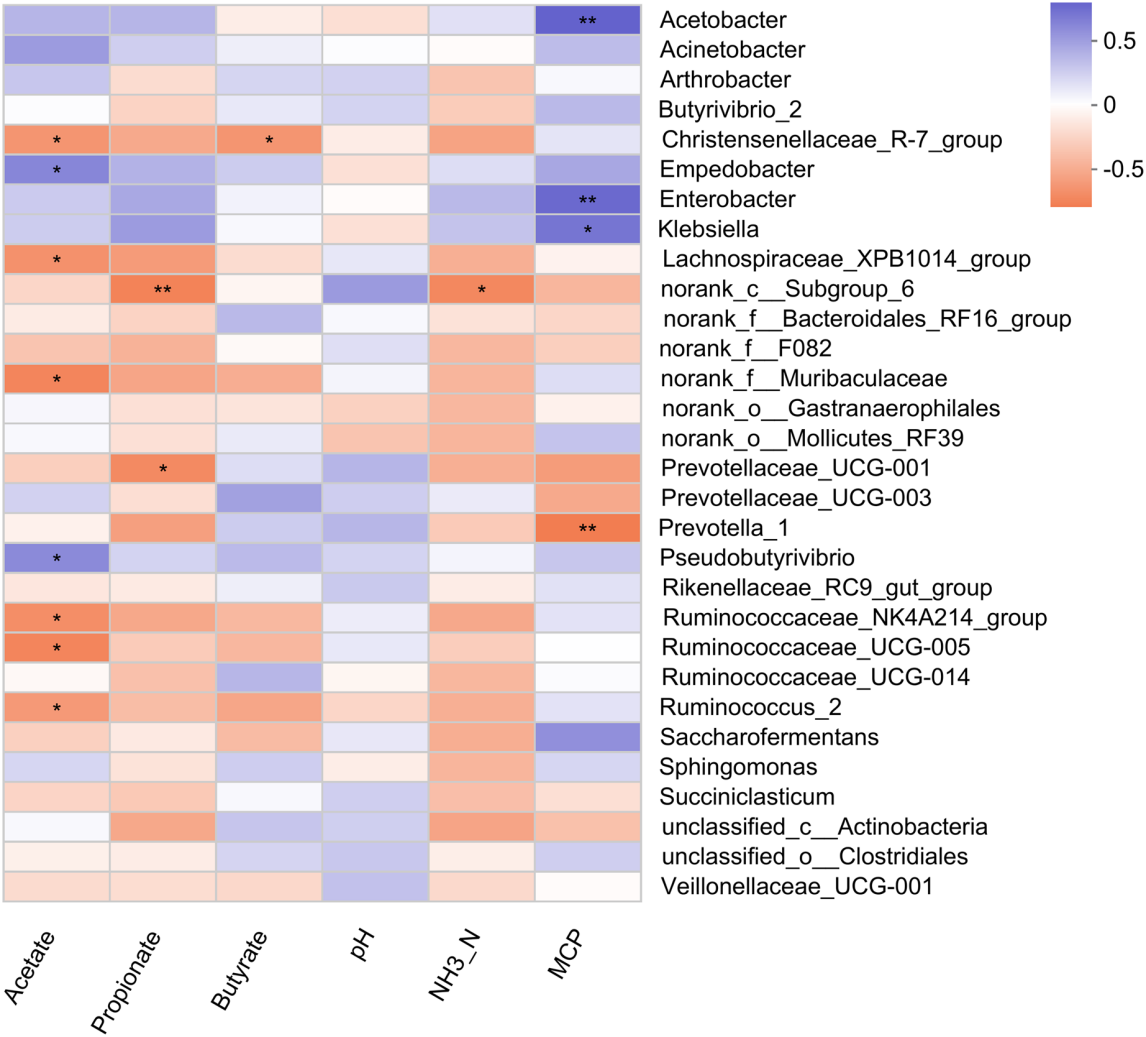
Correlation of rumen bacteria with fermentation parameters. Asterisks (* and **) indicate the significant correlation at *p* < 0.05 and *p* < 0.01, respectively.

Pearson correlation analysis revealed 5 positive and 11 negative correlations with rumen fermentation parameters (Fig. 4). A positive correlation of MCP was observed with three bacterial genera (Acetobacter, Enterobacter, and Klebsiella). The relative abundance of Empedobacter and Pseudobutyrivibrio also showed a positive correlation with the ruminal acetate. Six bacterial genera (Christensenellaceae_R-7_group, Lachnospiraceae_XPB1014_group, norank_f_Muribaculaceae, Ruminococcaceae_NK4A214_group, Ruminococcaceae_UCG-005, and Ruminococcus_2) showed a negative correlation with acetate, while two genera (Prevotellaceae_UCG-001, and norank_c_Subgroup_6) showed negative correlations with propionate. Only one bacterial genus showed a negative correlation with the rumen butyrate (Christensenellaceae_R-7_group) and NH_3_-N (norank_c_Subgroup_6) concentration.

### Association of rumen bacteria with milk yield parameters

**Figure 5:**
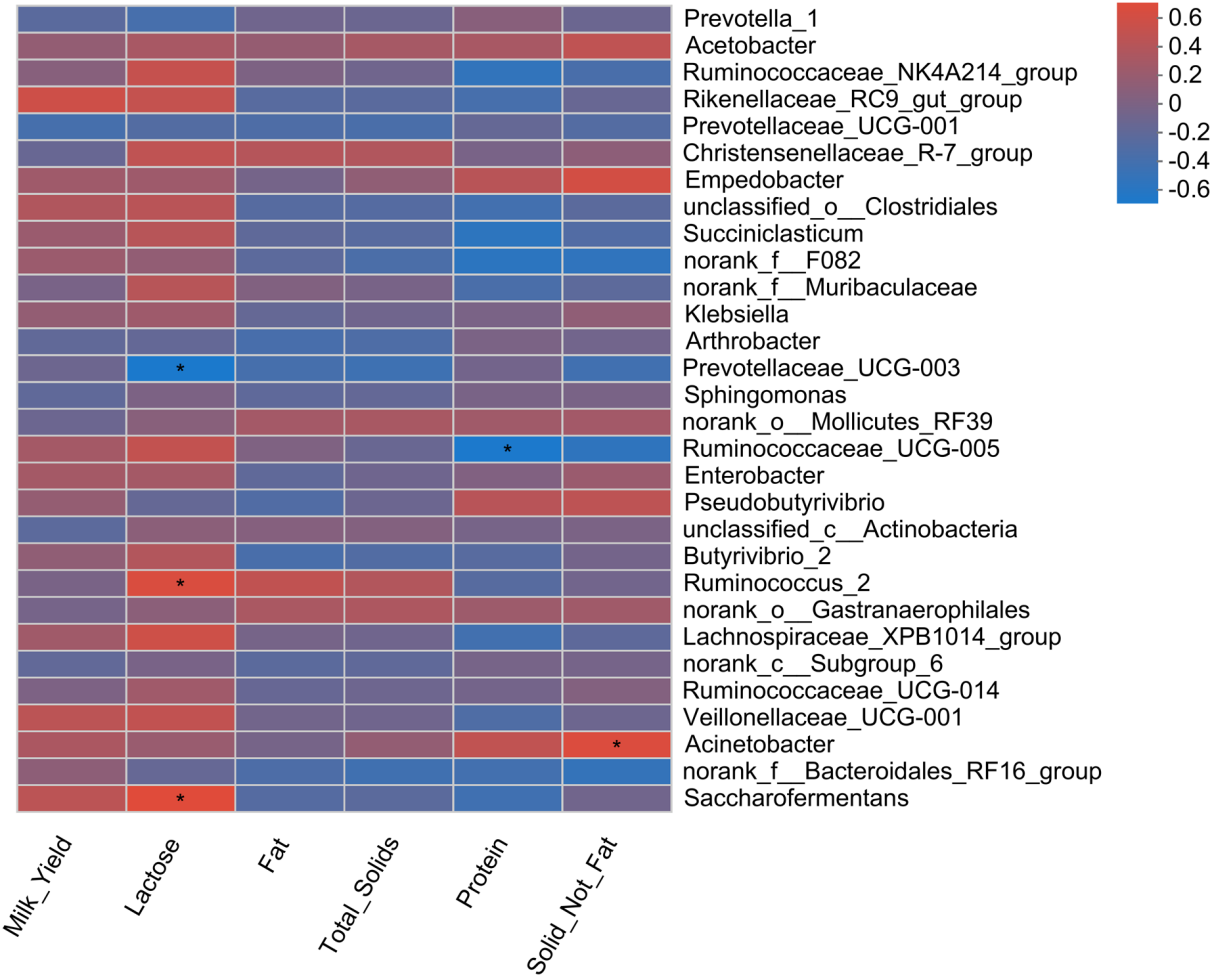
Correlation of rumen bacteria with milk yield and composition. Asterisks (*) indicate the significant correlation at *p* < 0.05, respectively.

Pearson correlation analysis revealed a total of four correlations, including two positive and two negative correlations, with milk yield parameters (Fig. 5). A positive correlation of two bacterial genera (Saccharofermentans and Ruminococcus_2) with milk lactose content was observed. Similarly, Acinebactor positively correlated with the solid not fat content of milk. Similarly, Ruminococcaceae_UCG005 showed a negative correlation with milk protein content. However, Prevotellaceae_UGC-003 showed a negative correlation with milk lactose content.

### Association of rumen bacteria with serum antioxidant parameters and heat shock proteins

**Figure 6:**
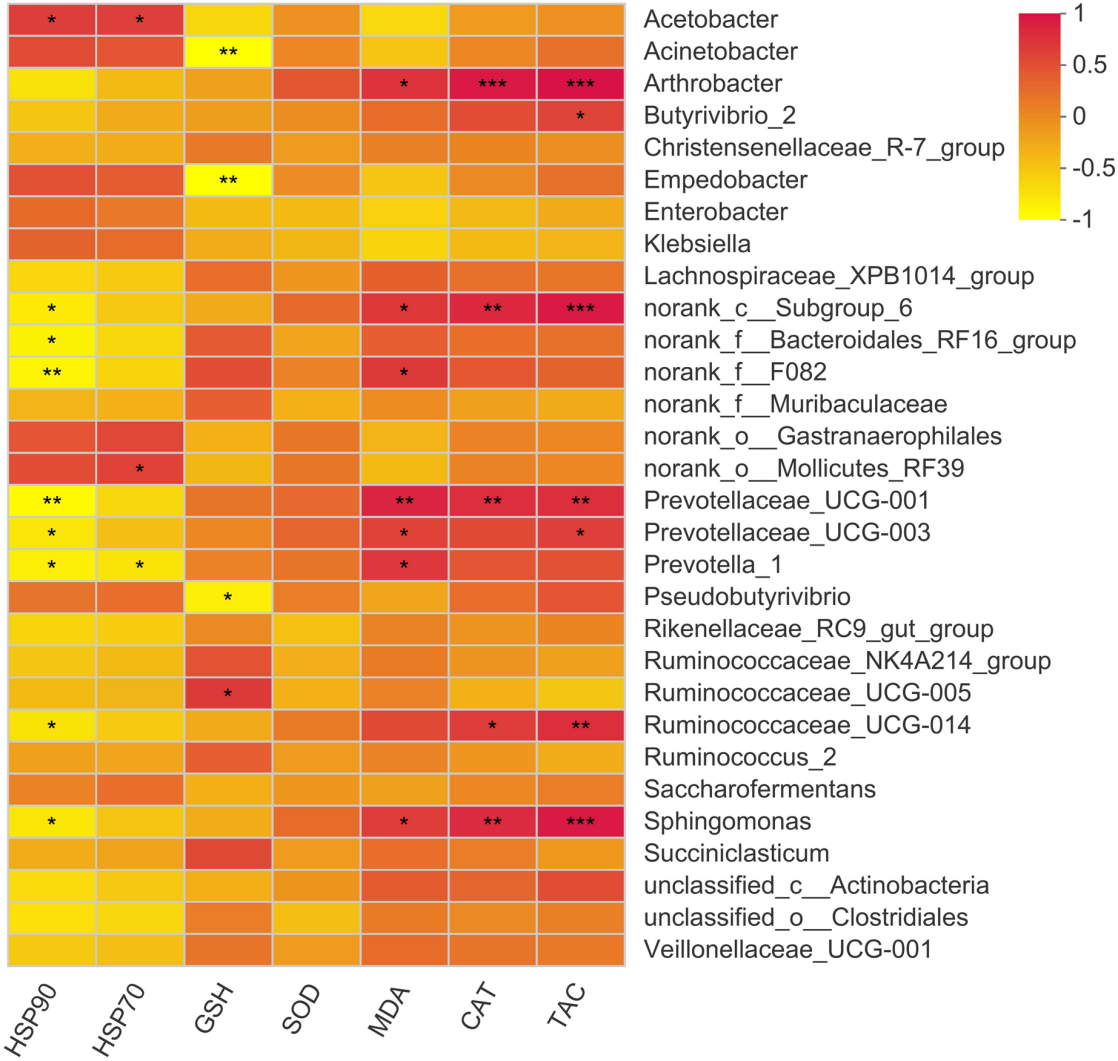
Correlation of rumen bacteria with serum antioxidant enzymes and heat shock proteins. Asterisks (*,** and ***) indicate the significant correlation at *p* < 0.05, *p* < 0.01 and *p* < 0.001, respectively.

The present study showed 23 positive and 12 negative correlations (*p* < 0.05) with bacterial genera (Fig. 6). *Acetobacter* showed a positive correlation with HSP70 and HSP90. The major genus *Prevotella* showed a positive correlation with MDA levels while showed a negative correlation with HSP70 and 90. Moreover, *Butyrivibrio* showed a positive correlation with serum T-AOC while *Arthobacter* showed a positive correlation with MDA, CAT, and T-AOC. Other correlations were mainly exhibited by bacterial genera with quite less abundance, so these are not important owing to their overall negligible effects on antioxidant status of the animal.

### Metagenomic prediction of functional genes and pathways of rumen bacteria

**Figure 7:**
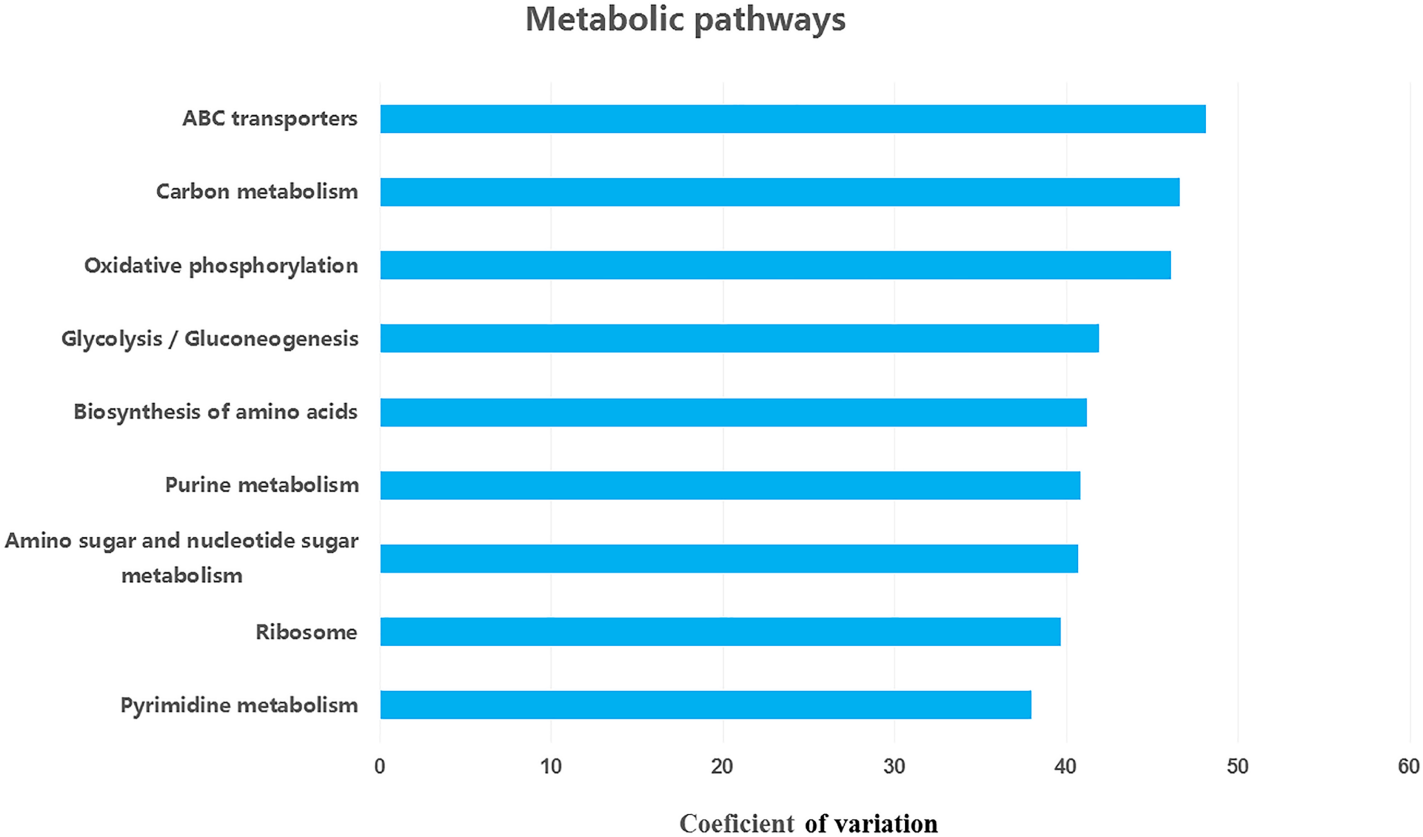
Top ten variable orthologs between control *vs*. treatment groups.

**Figure 8:**
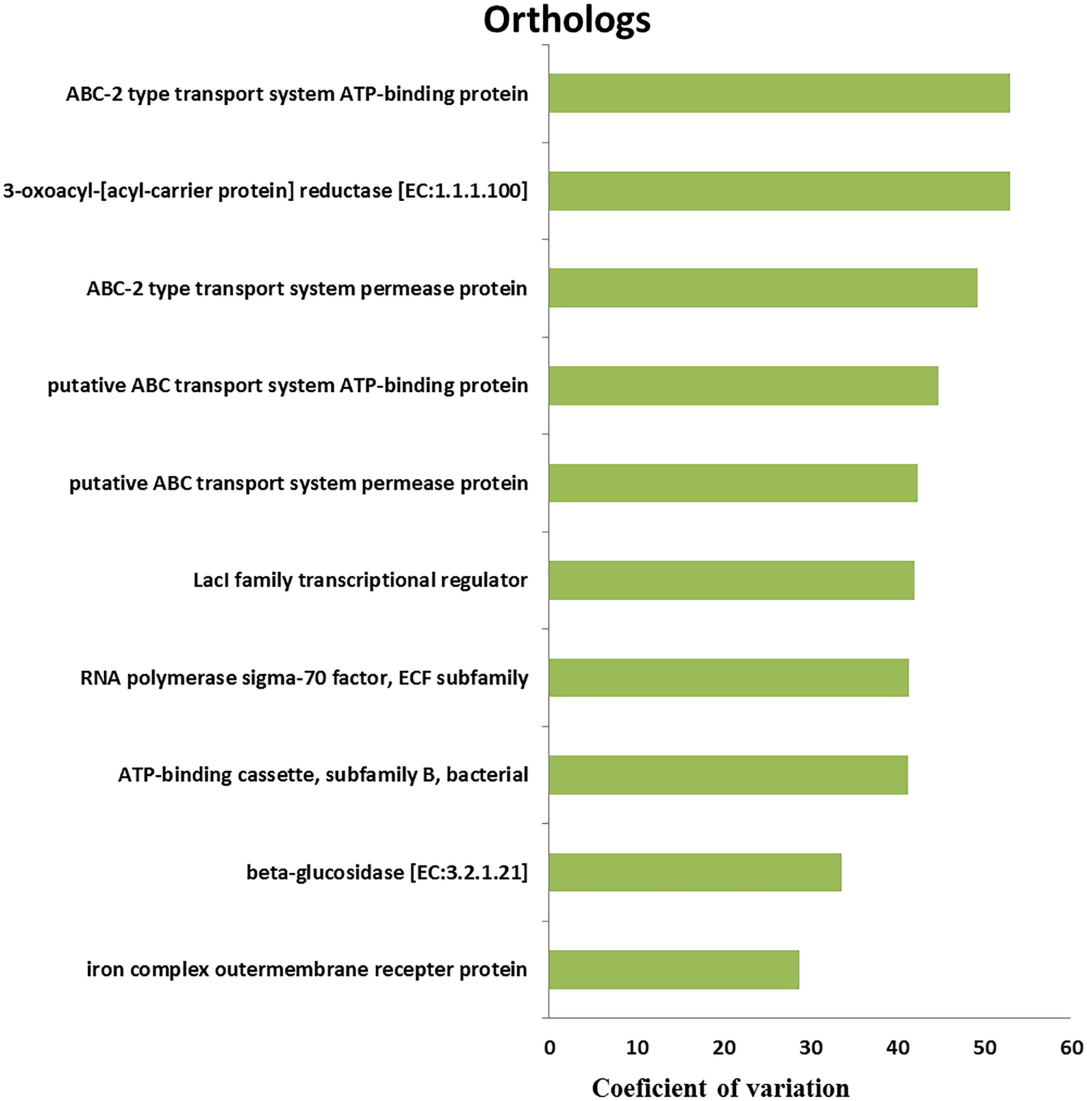
Top ten variable metabolic pathways in control *vs*. treatment groups.

We retrieved a total of 7,365 orthologous genes associated with 366 pathways in buffalo rumen microbiota. The most abundant genes and pathways (top 15) are presented in Table 3. The most abundant genes were the RNA polymerase sigma-70 factor, ECF subfamily, ABC-2 type transport system ATP-binding protein, and iron complex outer-membrane receptor protein (Table 3). The top three metabolic pathways included biosynthesis of amino acids, carbon metabolism, and ribosome. We calculated the coefficient of variation for each ortholog gene and metabolic pathway across different groups (control, MLF15, MLF30, and MLF45). The metabolic pathways and genes were sorted based on observed variation, and the top ten showing the largest variation among groups are presented in Figs. 7 and 8, respectively. The most variable genes were ABC-2 type transport system permease protein, 3-oxoacyl-[acyl-carrier protein] reductase [EC:1.1.1.100], and the ABC-2 type transport system ATP-binding protein and the most variable pathways involved ABC transporters, Carbon metabolism, and Oxidative phosphorylation. All these genes and pathways showed lower abundance in MLF groups as compared to the control.

**Table 3:** Top 15 (most abundant) genes and pathways from metagenomic prediction.

KeggID	Gene name	KeggID	Pathway
K03088	RNA polymerase sigma-70 factor, ECF subfamily	ko01230	Biosynthesis of amino acids
K01990	ABC-2 type transport system ATP-binding protein	ko01200	Carbon metabolism
K02014	iron complex outermembranerecepter protein	ko03010	Ribosome
K01992	ABC-2 type transport system permease protein	ko00230	Purine metabolism
K02004	putative ABC transport system permease protein	ko00240	Pyrimidine metabolism
K00059	3-oxoacyl-[acyl-carrier protein] reductase [EC:1.1.1.100]	ko02010	ABC transporters
K06147	ATP-binding cassette, subfamily B, bacterial	ko02024	Quorum sensing
K02003	putative ABC transport system ATP-binding protein	ko00190	Oxidative phosphorylation
K05349	beta-glucosidase [EC:3.2.1.21]	ko02020	Two-component system
K02529	LacI family transcriptional regulator	ko00520	Amino sugar and nucleotide sugar metabolism
K03086	RNA polymerase primary sigma factor	ko00010	Glycolysis/Gluconeogenesis
K01897	long-chain acyl-CoA synthetase [EC:6.2.1.3]	ko00620	Pyruvate metabolism
K06180	23S rRNA pseudouridine1911/1915/1917 synthase [EC:5.4.99.23]	ko00260	Glycine, serine and threonine metabolism
K03654	ATP-dependent DNA helicase RecQ [EC:3.6.4.12]	ko00970	Aminoacyl-tRNA biosynthesis
K01190	beta-galactosidase [EC:3.2.1.23]	ko01230	Biosynthesis of amino acids

## Discussion

### Rumen fermentation parameters

In the present study, we observed higher ruminal TVFA and propionate contents in the MLF45 group, which is consistent with earlier findings in Holstein calves fed with diets supplemented with MLF (Kong et al., 2019). A significantly higher concentration of rumen acetate in response to supplementation of MLF was observed, which is also previously reported in cattle as a result of feeding ensiled mulberry leaves (Zhou et al., 2014). Despite the higher acetate contents, MLF also proportionately increased the propionate contents to lower the acetate to propionate ratio, particularly with a dose of 45 g/d. The increase in propionate and reduction in acetate to propionate ratio observed in the present study is also consistent with previous studies involving dietary supplementation of flavonoids in Holstein heifers (Balcells et al., 2012). Similar findings have been reported in an *in vitro* study involving different pure flavonoids and their combinations (Seradj et al., 2014). In the MLF45 group, increased ruminal acetate content might be attributed to the higher abundance of Acetobactor, which is the main acetate producing bacteria in the rumen (Lyons et al., 2018). Acetobactor also plays a significant role in maintaining the ruminal anaerobic environment by consuming oxygen infused into the rumen during feeding, drinking, or rumination. This anaerobic environment facilitates the growth of anaerobic bacteria and archaea, subsequently leading to normal rumen functions. No effect of treatment was observed on rumen pH inspite of significant increase in acetate and TVFA contents which also has desirable effects as higher levels of VFAs can lower the rumen pH with negative consequences on rumen fermentation (Dijkstra et al., 2012). Moreover, optimum rumen pH and ecology improves energy intake and rumen fermentation, subsequently enhancing particularly propionate and MCP with positive impact on animal performance (Wanapat et al., 2014).

Flavonoids have shown to significantly increase in cellulolytic bacteria (*R. albus*) in the rumen of fattening steers fed with diets containing ensiled mulberry leaves (Li et al., 2017). Such shift in rumen microbiota can potentially enhance fiber digestion subsequently leading to enhanced rumen fermentation and VFA synthesis. Mulberry leaf and fruit pomace can enhance the volume of fermentable glucose and protein utilization by microorganisms subsequently leading to increase energy conversion efficiency of the rumen (Li et al., 2017; Zhou et al., 2012). Besides, mulberry flavonoids such as quercetin can effectively reduce the population of protozoa and methanogens subsequently leading to decrease methane emanation without negatively affecting rumen fermentation (Oskoueian, Abdullah & Oskoueian, 2013). Such decrease in protozoa and archeal community can consequently enhance growth of rumen bacteria through competitive exclusion (Ampapon, Phesatcha & Wanapat, 2019). Enhanced microbial turnover in the intestine can in turn lead to higher MCP content and N supply in the duodenal flow ultimately resulting in better nitrogen utilization efficiency (Balcells et al., 2012). It was evidenced by both significantly higher MCP and milk protein contents in the MLF45 group in the present study and our previous companion study (Li et al., 2020).

Energy metabolism under stressful conditions is a crucial consideration in ruminants to avoid the adverse effects of heat stress. In the present study, lower acetate to propionate ratio and higher TVFA content observed in MLF45 indicated the enhanced efficiency of rumen energy utilization, which facilitates mitigating the adverse effects of heat stress. Studies have reported that higher ruminal acetate content can decrease the efficiency of dietary energy utilization under heat stress conditions (Wheelock et al., 2010). On the other hand, propionate can enhance blood glucose levels through gluconeogenesis to address higher energy demands under heat stress conditions (Nafikov & Beitz, 2007; Abeni, Calamari & Stefanini, 2007). These findings suggest that MLF can effectively alleviate heat stress in buffalo without compromising the efficiency of dietary energy utilization by the rumen through desirably manipulating rumen bacteria. These findings indicated that mulberry flavonoids can desirably modulate rumen bacteriome to alter rumen fermentation subsequently leading to enhanced nutrient digestion, and VFA production in ruminants.

### Effect of MLF supplementation on rumen bacterial diversity

The present study revealed a significant decrease in the alpha diversity of rumen bacteria in response to MLF treatment. It may be attributed to well-known antimicrobial activities of flavonoids against some gram-negative and gram-positive bacteria as well as protozoa (Xie et al., 2015; de Aguiar Sílvia et al., 2013). Moreover, MLF have also shown to significantly reduce the pathogenic gut bacteria like E. Coli in calves indicating their toxic effects against certain gut microbes (Bi et al., 2017). The overall decrease in bacterial diversity is beneficial as it facilitates the evolution of a specific bacterial species and its dominance in the rumen ecosystem. In the present study, a decrease in bacteria diversity might be associated with depletion of Actinobacteria, Acidobacteria, Chlorofelxi, Patescibacteria, and Gemmatimonadetes in the MLF treated groups. It resulted in a comparatively high index of the dominance of rumen bacteria (Bacteriodetes and Firmicutes), which had positive influences on the host. Previous studies have also reported that increased bacterial diversity was not linked with improved growth performance, while decreased bacterial diversity resulted in a higher dominance index of Bacteroidetes (Prevotella_1), which subsequently led to better growth performance in sheep (Liu et al., 2019). Similar findings were obtained in our previous study as buffaloes exhibiting lower bacterial diversity (alpha diversity indices) showed superior performance in terms of milk yield, fat corrected milk, and milk protein content (Li et al., 2020). It may be attributed to the substantially higher dominance index of Acetobactor in the MLF45 group along with the lower Shannon index, bacterial richness, and evenness.

### Effect of MLF supplementation on the relative abundance of rumen bacteria

The overall abundance of Bacteriodetes increased in the present study, which is in agreement with previous studies reporting a positive effect of MLF on this bacterial phylum in calves (Nafikov & Beitz, 2007). As compared to the control, MLF15 and MLF30 numerically promoted the Prevotella up to 18 and 47%, respectively. The Prevotella was the major bacterial genus in the present study that accounted for 56.94% of total sequence reads. The Prevotella genus harbors various species of rumen bacteria that are specialized for fermenting starches, simple sugars, and other non-cellulosic polysaccharides (Purushe et al., 2010). Higher abundance of Prevotella observed in the buffalo rumen in the present study is consistent with previous findings reporting higher abundance of this genus in adult cattle exclusively in phylum Bacteriodetes as compared with calves fed with milk or milk replacer (Malmuthuge, Griebel & Guan, 2014; Jami et al., 2013). However, only MLF45 reduced the abundance of Prevotella as compared to the control (35.86% *vs*. 38.62%) in the present study. The association of a higher dose of MLF with decreased Prevotella shows the dose-dependent effect of flavonoids, which has also been reported earlier (Zhan et al., 2017).

Interestingly, treatment with MLF resulted in a 5.5 fold increase in the relative abundance of Acetobactor in the MLF45 group as compared to the other groups. No doubt, Acetobacter also increased in the MLF15 group (7.6% *vs*. 3.9%) and MLF30 group (4.5% *vs*. 3.9%) as compared to the control group, but this increase was negligible in comparison with abundance observed in the MLF45 group (21.5% *vs*. 3.9%). This increase in Acetobactor did not correspond with increasing levels of MLF as a medium (30 g/d) dose of MLF showed a lower abundance of these bacteria as compared with a lower dose (15 g/d). It is an important finding as it is a substantial increase in this genus comprising 21.5% of total bacterial sequences observed in the rumen contents. Studies have already reported a significant decrease in Acetobacter under heat stress conditions (Zhao et al., 2019). Similar findings were observed in our companion study as buffaloes in the control group showed higher values for all heat stress biomarkers (including MDA, CAT, SOD, and GSH-Px) as compared to the buffaloes supplemented with MLF45 owing to the excellent stress alleviation capability of MLF (Li et al., 2020). Thus our findings suggest that a substantial increase in Acetobactor was not only attributed to the direct effect of MLF, but it was partially attributed to the alleviation of heat stress that also contributed to the increase in the abundance of Acetobactor in the rumen of MLF supplemented buffaloes. To corroborate these findings, we also calculated the correlation of serum antioxidant enzymes and heat shock protein contents with bacteria genera, which revealed positive correlation of Acetobactor with serum HSP70 and 90 contents.

Four bacterial genera, including norank_f__F082, Ruminococcaceae_UCG-005, norank_f__Bacteroidales_RF16_group and Anaerovibrio specifically showed relatively higher abundance in the MLF15 group as compared to other groups. Interestingly, a significant increase of Pseudobutyrivibrio was only observed in the MLF45 group, revealing a favorable effect of a high dose of flavonoids on this bacterial genus. A previous *in vitro* study has shown that Butyrivibrio species can use rutin as an energy source (Leedle & Hespell, 1980), which explains why its higher abundance was observed only in maximum dose of the MLF group (45 g/d) in the present study.

### Association of rumen bacteria with rumen fermentation, milk yield, and serum antioxidant parameters

The present study revealed a positive correlation of MCP with three bacterial genera (Acetobacter, Enterobacter, and Klebsiella). The positive correlation of Empedoobacter and Pseudobutyrivibrio with ruminal acetate concentration was well associated with higher acetate content observed in MLF45. Moreover, bacteria belonging to the Ruminococcaceae family (Ruminococcaceae_NK4A214_group, Ruminococcaceae_UCG-005, and Ruminococcus_2) are the major cellulolytic bacteria that produce propionate. That’s why their higher abundance favored the production of propionate while suppressed the ruminal acetate content as they showed negative correlation with acetate [59].

Enhanced milk yield and milk protein content for MLF treated buffalos might be attributed to higher TVFA and MCP (Li et al., 2020). Moreover, these desirable effects on productive performance may be attributed to enhanced nutrient digestion and absorption in response to MLF supplementation. Because flavonoid aglycones have been suggested to interact with mucin (*via* non-covalent interactions) that protects the mucus layer and serve as a selective barrier during nutrient digestion and absorption (Gonzales et al., 2016). Due to this ability, MLF have shown to lower the thickness of abomasal and duodenal mucosa in calves that resulted in the more significant absorption of nutrients. Meanwhile, it reduced the absorption of toxins and altered the blood metabolites, fecal microbial content, and animal performance (Wang et al., 2017). It is pertinent to mention that rumen epithelium harbors numerous papillae that are mainly responsible for significant activities of nutrient absorption and utilization (Graham & Simmons, 2005). It is attributed to the fact that MLF can mediate the morphology of the rumen and GIT as both of their structures are highly sensitive to dietary physical or chemical changes even in adult lactating dairy cows (Wang et al., 2017). We did not measure the nutrient digestibility in the present study, so we cannot corroborate these findings directly, which is a limitation of this study and should be accounted for in future studies.

The gut microbiome is not only involved in nutrient digestion and utilization but also serve as a significant mediator of metabolic and immune health. Its role becomes even more crucial under stressful conditions such as metabolic disorders (acidosis) and heat stress. The putative mechanism of antioxidant effects of rumen microbiota involves the chelation of metal ions, production of own antioxidant enzymes, antioxidant metabolites, upregulation of antioxidant capacity, and enhancement of host antioxidant metabolites. Moreover, they coordinate the different signaling pathways, mediate activities of reactive oxygen species, and enrich lower gut microbiota (Wang et al., 2017). A recent study has shown an increase in Firmicutes and Lactobacillus after exposure to transportation stress in cattle Yak (Li et al., 2019). The present study was conducted during the summer season under the hot and humid climate of South China with an average Temperature Humidity Index (THI) of 82. A higher level of MLF substantially decreased the oxidative stress marker (MDA) by 75% and Glutathione peroxidase (GSH-Px) as compared to the control group (Li et al., 2020). The present study showed 23 positive and 12 negative correlations (*p* < 0.05) of rumen bacteria with different antioxidant parameters. The positive correlation of Acetobacter was observed with serum heat shock proteins (HSP70 and HSP90). Our findings regarding the positive correlation of Prevotella with MDA levels and negative correlation with HSP70 and 90 are also consistent with earlier studies reporting negative correlation of Prevotella with antioxidant status (serum BHBA) in dairy calves (Meale et al., 2017). Moreover, a positive correlation of Butyrivibrio with serum total antioxidant capacity is also in line with earlier findings of Li et al. (2019), about the negative correlation of this bacterial genus with serum anti-inflammatory cytokines. They reported that relative abundance of Butyrivibrio 2 showed a negative correlation with serum IL-4 and IL-10 and positive correlation with serum lactic acid (R = 0.561, *p* < 0.01).

The association of rumen bacteria with serum antioxidant capacity observed in the present may be attributed to the metabolites produced by rumen bacteria, which might serve as postbiotics. Dietary postbiotics have shown to enhance the antioxidant capacity of serum and rumen fluid while reducing the serum lipid peroxidation and upregulating the hepatic antioxidant enzymes and ruminal barrier function (Izuddin et al., 2020).

### Metagenomic prediction and functional profiling

The present study revealed that the most variable genes belonged to the ABC membrane transport system permease protein (which is the member of a superfamily of transmembrane proteins universally present in all organisms from prokaryotes to mammals), carbon metabolism, and oxidative phosphorylation. The carbon metabolism (responsible for hydrolysis, fermentation, VFA oxidation, and methanogenesis) is the major activity performed in the rumen during the degradation of plant biomass. Reduction of overall carbon metabolism can favor a decrease in VFA oxidation and CH_4_ production that can subsequently increase the energetic efficiency of the host (Andersen et al., 2020). Moreover, third most variable pathway was oxidative phosphorylation, which is the primary process for energy production in the mitochondria of microbes but generates reactive oxygen species that can increase oxidative stress and initiate an antioxidant response, which compromises cell survival and its putative functions (Landolfo et al., 2008). It is beneficial in terms of reducing oxidative stress in microbial cells to favor their metabolic activities and putative functions. The oxidative stress caused by oxidative phosphorylation interferes with both protein folding and membrane translocation (Costa, Quintanilha & Moradas-Ferreira, 2007) and reduces the expression of glycolytic enzymes (Hamm-Künzelmann et al., 1997). Overall, the decrease observed in the enrichment of orthologs and pathways in response to MLF treatment is mainly attributed to the antimicrobial effects imparted by MLF.

Our study demonstrated that MLF could substantially improve the rumen fermentation by mediating the rumen microbiome through decreasing bacterial diversity and promoting specific species like norank_f__F082, Acetobactor and Empedobactor. A significant increase in TVFA, acetate, propionate, acetate: propionate, and MCP observed in the MLF45 group indicate that 45 g/d is the optimal dose of MLF for supplementation. This group also showed significantly higher milk yield, fat corrected milk, and milk protein (%) as compared to other groups (Li et al., 2020). These desirable effects of MLF on rumen fermentation are mediated through two ways; (1) directly affecting the rumen microbiota and their functional profile, (2) alleviating heat-induced oxidative stress in buffalo that subsequently affect rumen microbe (oxidative phosphorylation) and nutrient metabolism through mediating glucose and fatty acid metabolism. It is well established that heat stress can decrease the TVFA, acetate, NH_3_-N, and acetate to propionate ratio in ruminants (Cai et al., 2019; Yadav, 2013). Such adverse effects may be mediated by reducing feed intake and adversely affecting nutrient metabolism by altering the rumen microbiome. So alleviation of heat stress might have desirable effects on these rumen fermentation parameters, as observed in the present study. Therefore, our findings indicate that dietary supplementation of MLF can improve the rumen fermentation and nutrient digestibility by positively affecting the rumen microbiome, particularly under heat stress conditions of tropical climates. However, further studies are required to corroborate these findings by investigating larger cohorts to evaluate effect of MLF on rumen metabolome, nutrient digestion and metabolic pathways particularly glucose and fatty acid metabolism.

## Conclusions

Dietary supplementation of mulberry flavonoids significantly increased the TVFA, acetate, propionate, acetate to propionate ratio, NH_3_-N, and microbial protein. Treatment significantly decreased the bacterial alpha diversity parameters except the Simpson index. Mulberry flavonoids favored Bacteriodetes, Firmicutes, and Proteobacteria while suppressed Actinobacteria, Acidobacteria, Chloroflexi, Patescibacteria, and Gemmatimonadetes. A substantial increase in Acetobactor was observed with addition of mulberry flavonoids at 45 g/d that positively affected the rumen fermentation parameters. Moreover, mulberry flavonoids promoted the Empedobactor, Klebsiella, and Pseudobutyrivibrio in the rumen. The findings of the present study indicated that 45 g/d is an appropriate dose for dietary supplementation in buffaloes to desirably enhance rumen fermentation and bacteriome for better performance under hot and humid climate.

## Supplemental Information

10.7717/peerj.14309/supp-1 Supplemental Information 1 Supplementary Tables.

10.7717/peerj.14309/supp-2 Supplemental Information 2 Raw Data for Table 1.

10.7717/peerj.14309/supp-3 Supplemental Information 3 The ARRIVE guidelines 2.0: author checklist.

## Additional Information and Declarations

### DNA Deposition

The following information was supplied regarding the deposition of DNA sequences: The raw 16-S RNA gene sequences are available at SRA NCBI: PRJNA680172 and SRR13167472.

### Data Availability

The following information was supplied regarding data availability: The raw data are available in the Supplemental File.
