# Supplementary material for: Mulberry flavonoids modulate rumen bacteria to alter fermentation kinetics in water buffalo
Source: PeerJ. 2022 Dec 14;10:e14309. doi: 10.7717/peerj.14309 (PMC9758972; doi:10.7717/peerj.14309)
Supplement: Supplemental Information 1 [file peerj-10-14309-s001.docx]

**Table S-1 Composition and nutrient levels of basal diets (Air-dry basis, %)**

| Items | Content |
| --- | --- |
| Ingredients |  |
| *Pennisetumpurpureumschum* | 32.82 |
| Brewer’s grain | 48.28 |
| Corn | 9.44 |
| Wheat bran | 3.98 |
| Soybean meal | 3.03 |
| Limestone | 0.26 |
| NaCl | 0.37 |
| CaHPO_4_ | 0.32 |
| CaHPO_4_ | 0.42 |
| Premix^1^ | 0.18 |
| Total | 100 |
| Nutrient level^2^ |  |
| CP | 15.23 |
| NDF | 39.57 |
| ADF | 27.98 |

^1^The premix provided the following per kg of diets：VE 3 000 IU，VD 150 000 IU，VA 500 000 IU，Cu 1.3 g，Fe 4.0g，Mn 3.0g，I 80 mg，Zn6.0 g，Co 80 mg，Se 50 mg.

^2^Measured values.

Table S-2. Relative abundance of bacterial phyla in different treatment groups (% of sequences)

| **Phylum name** | **Control** | **MLF15** | **MLF30** | **MLF45** | **P value** |
| --- | --- | --- | --- | --- | --- |
| Bacteroidetes | 49.11 | 60.21 | 69.09 | 48.01 | 0.161 |
| Proteobacteria | 14.97 | 11.85 | 7.59 | 27.80 | 0.066 |
| Firmicutes | 14.01 | 24.43 | 20.32 | 18.88 | 0.282 |
| Actinobacteria | 9.91 | 0.23 | 0.22 | 0.30 | 0.066 |
| Acidobacteria | 2.84 | 0.00 | 0.00 | 0.00 | 0.024 |
| Chloroflexi | 2.82 | 0.00 | 0.00 | 0.01 | 0.059 |
| Patescibacteria | 1.41 | 0.39 | 0.48 | 0.30 | 0.094 |
| Gemmatimonadetes | 0.93 | 0.00 | 0.00 | 0.00 | 0.025 |
| unclassified_k__norank_d__Bacteria | 0.81 | 0.14 | 0.14 | 0.17 | 0.099 |
| Cyanobacteria | 0.52 | 0.40 | 0.43 | 1.23 | 0.740 |
| Spirochaetes | 0.43 | 0.84 | 0.67 | 0.27 | 0.057 |
| Tenericutes | 0.40 | 0.85 | 0.36 | 2.39 | 0.183 |
| Rokubacteria | 0.33 | 0.00 | 0.00 | 0.00 | 0.013 |
| Lentisphaerae | 0.22 | 0.21 | 0.28 | 0.19 | 0.679 |
| GAL15 | 0.17 | 0.00 | 0.00 | 0.00 | 0.013 |
| Nitrospirae | 0.15 | 0.00 | 0.00 | 0.00 | 0.013 |
| Latescibacteria | 0.12 | 0.00 | 0.00 | 0.00 | 0.013 |
| Deinococcus-Thermus | 0.06 | 0.00 | 0.00 | 0.00 | 0.042 |
| Elusimicrobia | 0.03 | 0.01 | 0.03 | 0.22 | 0.022 |
| Synergistetes | 0.02 | 0.07 | 0.08 | 0.02 | 0.040 |

Table S-3. Relative abundance of bacterial genera in different treatment groups (% of sequences)

| **Genus name** | **Control** | **MLF15** | **MLF30** | **MLF45** | **P value** |
| --- | --- | --- | --- | --- | --- |
| Prevotella_1 | 38.62 | 45.58 | 56.94 | 35.86 | 0.161 |
| Acetobacter | 3.93 | 7.59 | 4.54 | 21.53 | 0.070 |
| Arthrobacter | 1.96 | 0.00 | 0.01 | 0.03 | 0.024 |
| Prevotellaceae_UCG-001 | 1.95 | 2.27 | 2.38 | 1.49 | 0.183 |
| Rikenellaceae_RC9_gut_group | 1.50 | 3.31 | 2.13 | 2.07 | 0.066 |
| Sphingomonas | 1.49 | 0.07 | 0.06 | 0.18 | 0.043 |
| unclassified_c__Actinobacteria | 1.38 | 0.00 | 0.00 | 0.00 | 0.013 |
| Ruminococcaceae_NK4A214_group | 1.31 | 3.95 | 2.75 | 1.32 | 0.147 |
| Empedobacter | 1.29 | 0.73 | 1.23 | 4.01 | 0.070 |
| Christensenellaceae_R-7_group | 1.27 | 2.17 | 2.51 | 1.97 | 0.270 |
| norank_c__Subgroup_6 | 1.21 | 0.00 | 0.00 | 0.00 | 0.025 |
| norank_f__F082 | 1.18 | 2.11 | 1.76 | 0.76 | 0.038 |
| unclassified_o__Clostridiales | 1.14 | 2.67 | 1.27 | 1.04 | 0.270 |
| norank_o__Saccharimonadales | 0.83 | 0.00 | 0.00 | 0.00 | 0.013 |
| Nocardioides | 0.78 | 0.00 | 0.00 | 0.00 | 0.025 |
| Pseudobutyrivibrio | 0.55 | 0.24 | 0.18 | 1.24 | 0.029 |
| norank_f__Bacteroidales_RF16_group | 0.41 | 1.06 | 0.33 | 0.05 | 0.022 |
| Acinetobacter | 0.35 | 0.17 | 0.33 | 1.13 | 0.041 |
| Klebsiella | 0.35 | 1.39 | 0.78 | 1.49 | 0.092 |
| Ruminococcaceae_UCG-005 | 0.27 | 1.17 | 0.95 | 0.36 | 0.034 |
| Anaerovibrio | 0.19 | 0.51 | 0.38 | 0.10 | 0.033 |
| Comamonas | 0.13 | 0.18 | 0.21 | 0.31 | 0.050 |
| Stenotrophomonas | 0.11 | 0.32 | 0.30 | 0.49 | 0.043 |
| Prevotellaceae_NK3B31_group | 0.10 | 0.16 | 0.39 | 0.10 | 0.038 |
| Ruminococcaceae_UCG-005 | 0.27 | 1.17 | 0.95 | 0.36 | 0.034 |
